# Saloon Door Technique – “open sky” IOL exchange utilising flanged haptic fixation behind a pre-existing Artificial Iris

**DOI:** 10.1016/j.ajoc.2025.102431

**Published:** 2025-09-10

**Authors:** Kilian Roth, Konstantin Seiller-Tarbuk, Michael Amon

**Affiliations:** a Department of Ophthalmology, Hospital of the Brothers of St. John of God Vienna, Johannes von Gott Platz 1, 1020, Vienna, Austria; b Sigmund Freud Private University Vienna, Campus Prater, Freudplatz 1+3, 1020, Vienna, Austria

**Keywords:** Artificial Iris, IOL exchange, Flanging technique, Yamane technique, Saloon Door Technique, Forceps needle

## Abstract

**Purpose:**

This paper introduces a novel surgical approach, the “Saloon Door Technique”, which allows intraocular lens (IOL) replacement behind a pre-existing Artificial Iris (AI) without its removal.

**Observations:**

A 57-year-old patient with a history of complicated cataract surgery, AI implantation, and recurrent corneal decompensation presented with a dislocated anterior chamber IOL. To prevent further corneal decompensation, we performed an IOL exchange and penetrating keratoplasty under open-sky conditions while preserving the AI. The AI was radially incised at two opposite sites to allow the insertion of a new 3-piece IOL through the ‘saloon door’ into the posterior chamber. The haptics were then externalized and flanged with a Forceps Needle using a modified Yamane technique. The postoperative course was uneventful, with stable IOL positioning and a clear corneal graft at follow-up.

**Conclusions and importance:**

The Saloon Door Technique enables IOL implantation behind a pre-existing AI while preserving its integrity. By making two controlled incisions in the AI, the IOL can be carefully maneuvered through the “saloon door”, followed by scleral fixation using a modified Yamane technique. This technique represents a valuable advancement in anterior segment surgery for complex cases involving AI and IOL exchange.

## 1 Introduction

The anterior segment of the eye is extremely complex, as many fundamentally different structures come together in a very small space. This results in a wide variety of surgical techniques for the respective affected structures. Several fundamentally different structures can often be affected simultaneously in the anterior segment of the eye, or the causally affected structure and the resulting surgical consequences can have an impact on the neighboring structures and vice versa.

In recent years, flanging techniques have revolutionized IOL fixation by offering sutureless methods that reduce suture-related complications. First pioneered by Yamane, the technique employs a double-needle approach to externalize haptics and create stable fixation by cauterizing the ends to form flanges.^1^ Subsequent modifications, such as the double flange method of suture fixation introduced by Canabrava, have expanded the scope of these innovative methods and provide surgeons with further solutions to the individual challenges of anterior segment surgery.^2^

In our patient, several problems came together. Due to a causative complicated cataract operation, the patient received an AI. Subsequently, a dislocated anterior chamber lens led to recurrent corneal decompensation resulting in multiple corneal surgeries.

In this case, we present a novel approach for the explantation of a dislocated AC-IOL and implantation of a new 3-piece-IOL with a pre-existing AI and leaving it in place. In our technique, the AI was preserved under open-sky conditions in the context of a penetrating keratoplasty and radially incised at two sites (180° to each incision) to insert an IOL through “a saloon door” into the posterior chamber. The haptics were then externalized and flanged using a modified Yamane technique to ensure secure scleral fixation behind the AI. This approach avoids the need to remove the AI and minimizes trauma while achieving a stable, cosmetically unobtrusive and functionally good result.

## 2 Case report

A 57 years old lady had a complicated cataract surgery resulting in aphakia and aniridia in 1990 at her right eye. Because of contact-lens intolerance in 1998 a black iris-diaphragm intraocular lens (ID-IOL) was sutured with 10.0 prolene suture to the sclera. After ID-IOL dislocation in 2008, an AI and anterior chamber polymethylmethacrylate (PMMA) IOL were implanted. Although surgical records from the original procedure were unavailable due to it being performed at an external institution, preoperative slit-lamp examination strongly suggested that the AI was a model by HumanOptics (HumanOptics AG, Erlangen, Germany), based on its characteristic appearance. In consideration of the fact that HumanOptics manufactures both fiber-containing and fiber-free models, intraoperative inspection confirmed the presence of polymer fibers within the implant in this case. Between 2009 und 2023 recurrent corneal decompensations caused three descemet membrane endothelial Keratoplasty (DMEK) and two penetrating Keratoplasty (PK) procedures. In 2023, the patient was referred because of the next corneal decompensation.

At the subsequent presentation in our hospital the AI was in place from 15 years with unknown dimension of the prolene sutures and unknown fixation site. The type of the PMMA IOL was not detectable. The patient's vision was limited to finger counting. Amblyopia in the affected eye was confirmed in the anamnesis.

**Fig. 1:**
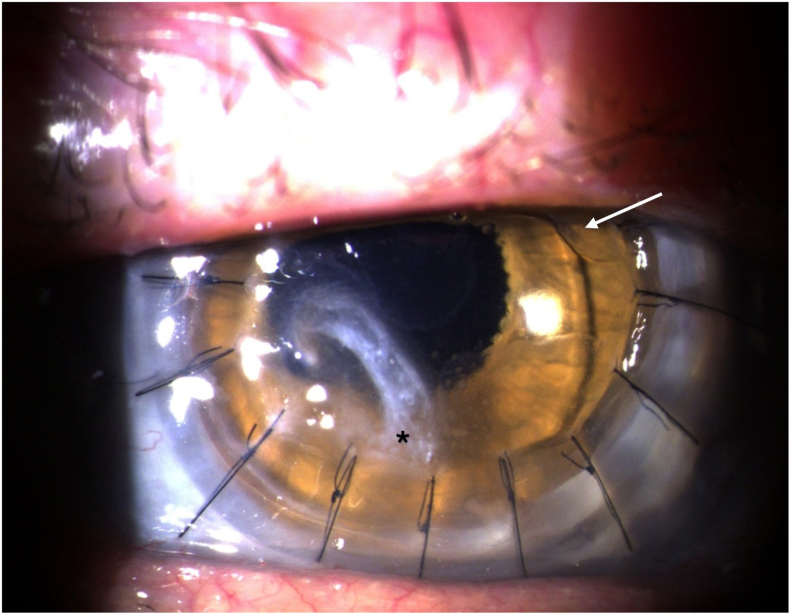
Pre-surgery slit lamp photo of the right eye. White arrow shows the IOL. Black asterisk shows the corneal edema.

Slit lamp examination revealed total corneal edema, with the most prominent edema in the mid-peripheral area around the 7–8 o'clock position and a simultaneous thinning of the donor tissue at the interface at 7 o'clock (Fig. 1). Giant cells on the front surface of the IOL were observed. The radially arranged single-button sutures were tight and in place from the previous PK procedure. Anterior chamber OCT (AC-OCT) showed an IOL in the anterior chamber with light contact to the corneoscleral interface. The AI was well centered and in place. The posterior segment of the eye could only be vaguely assessed due corneal decompensation. As far as can be assessed, this appeared inconspicuous. A posterior vitrectomy was known to have been performed. The ultrasound examination showed an attached retina.

Together with the patient, it was decided to replace the dislocated IOL — as it was suspected to be largely responsible for the recurrent corneal decompensations — and to perform a new PK. The decision to implant a new IOL was based on the expectation of residual visual potential: the patient's vision was limited to finger counting, and amblyopia in the affected eye was confirmed in the anamnesis; however, the patient recalled previously being able to read large print with the eye. Due to corneal decompensation, no reliable refraction or contact lens trial was feasible preoperatively. In light of the patient's contact lens intolerance and desire for visual improvement, IOL implantation was deemed a reasonable attempt to restore some functional vision.

## 3 Methods

• **Surgical rationale**

Our assumption was that the dislocated IOL in the anterior chamber was the primary cause of corneal decompensation, necessitating the fixation of a new IOL behind the AI to minimize the risk of future corneal complications. The goal was to address all of these issues in a single surgical session, involving keratoplasty and IOL (+ AI ?) fixation, to minimize surgical trauma. However, there were two options, both of which would result in the loss of the currently well-fitting AI:

• Option 1: Removal of both the IOL and AI, followed by combined fixation of the iris prosthesis and the IOL using a modified Yamane or Canabrava flanging technique.^3^^,^^4^
• Option 2: Removal of both the IOL and AI, followed by separate scleral fixation of the AI and of the IOL.

Both options were considered, but neither was deemed perfect, as both would result in the loss of the AI. To preserve the AI and make the procedure more atraumatic, we developed a new technique, the Saloon Door Technique, to fixate an IOL behind the preexisting AI by performing two radial cuts at the AI to allow the IOL to pass through this opening, and then scleral fixating the IOL using a modified Yamane technique.

• Surgical technique step-by-step

The procedure began with the excision of the decompensated donor cornea. The cornea was first marked and then trephined with a Hessburg trephine to create a circular incision. This established an open-sky setting.

The IOL in the anterior chamber was then visualized, with particular adhesion noted between the inferior haptic and the cornea. This adhesion was carefully prepared using corneal scissors and a spatula.

Next, the AI was radially incised at the 12 o'clock and 6 o'clock positions. The 3-piece IOL used for implantation was a foldable acrylic lens, the Kowa PU6AS (Kowa Company, Ltd., Japan), which is suitable for scleral fixation using the modified Yamane technique. Before tunnel creation, scleral marks were placed at the 2 o'clock and 8 o'clock positions, approximately 2 mm posterior to the limbus. A Forceps Needle^5^ was then used to prepare the first scleral tunnel, a step complicated by the pre-existing vitrectomy and the open-sky setting. The haptic was externalized using the Forceps Needle (Geuder AG, Heidelberg, Germany) and fixated with flanging according to the modified Yamane technique.

**Fig. 2:**
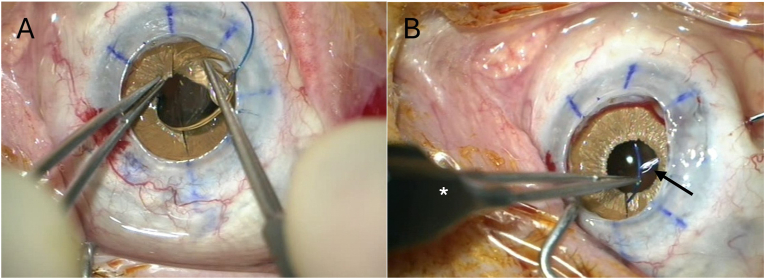
a,b. intraoperative implantation. Fig. 2a. IOL maneuverer through the incised AI in the posterior chamber. Fig. 2b. From the left in the picture the forceps (white asterisk) holding the haptic to be externalized and from the right in the picture the Forceps Needle (black arrow) with its open gripper arms.

The IOL was carefully maneuvered through the incised AI, referred to as the “saloon door,” into the posterior chamber (Fig. 2a). During this process, the opposite haptic remained in the anterior chamber. The second scleral tunnel was then prepared using the Forceps Needle (Fig. 2b). This step was particularly very challenging due to the lack of intraocular pressure and required multiple instances of globe stabilization with balanced salt solution (BSS) to restore adequate tone. Once completed, the second haptic was externalized and flanged.

Intraoperatively, due to the open-sky setting and the patient's history of pars plana vitrectomy, no posterior trocar was placed. Instead, globe stability was maintained by manually injecting balanced salt solution (BSS) into the posterior chamber. This approach prevented uncontrolled and potentially suboptimal fluid flow from the posterior chamber into the anterior chamber and out of the eye, which might otherwise occur with posterior infusion under open-sky conditions.

Finally, the corneal transplantation was performed, with the new donor cornea secured using 16 interrupted sutures. The procedure concluded without complications, resulting in a well-toned globe at the end of surgery.

The surgery was performed by M.A. and the surgical video is attached in the appendix. (Video 1).

Supplementary data related to this article can be found online at https://doi.org/10.1016/j.ajoc.2025.102431

The following are the Supplementary data related to this article:

Video 1 Saloon Door Technique. Video 1

## 4 Results

The postoperative course was uneventful. The patient was discharged the day after surgery in good condition. Intraocular pressure remained stable (<20 mmHg) throughout all follow-up visits.

**Fig. 3:**
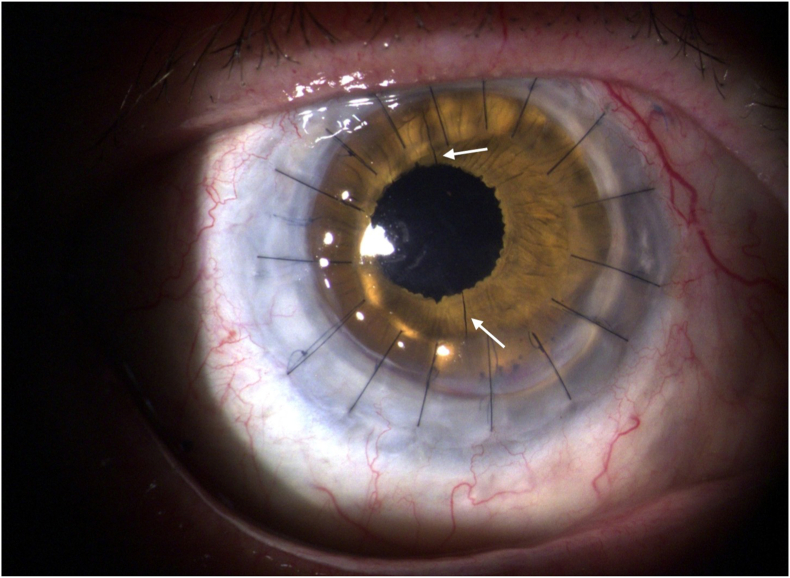
Post-surgery slit lamp photo of the right eye. White arrows shows the incisions of the AI.

By the one-month follow-up, anterior chamber bleeding had resolved, and the donor cornea appeared clear, with secure sutures and well-centered IOL positioning. A mild step formation was observed at the graft-host junction (5–7 o'clock), which remained stable through month nine. The scleral flanges at 2 and 8 o'clock were conjunctivally covered and remained in position without signs of erosion (Fig. 3).

Visual acuity improved progressively: uncorrected visual acuity (UDVA) at one month was +1.30 logMAR, and best-corrected visual acuity (CDVA) reached +0.80 logMAR at nine months (+2.00 sph, +2.00 cyl at 150°). Corneal sutures were still in place at the last visit; removal was planned at the upcoming one-year follow-up.

Topical therapy included a standard steroid taper with dexamethasone eye drops, adjunctive steroid ointment (prednisolone pivalate), antibiotics (ofloxacin), and lubricants. No systemic immunosuppression was administered, as per institutional protocol for non-autoimmune cases.

A detailed timeline of medication is provided in (Supplementary Table 1).

## 5 Discussion

A key consideration in this case was the necessity of explanting the anterior chamber IOL, as we strongly suspected it to be the primary cause of corneal decompensation. During surgery, it was observed that the inferiorly positioned haptic had grown into the cornea, which likely contributed to the endothelial failure. This finding also correlates with the presence of foreign-body giant cells observed during slit-lamp examination, further supporting our preoperative suspicion of chronic endothelial stress caused by direct IOL contact.

Furthermore, ensuring optimal conditions for the acceptance of the donor cornea was a crucial objective. Any persistent contact between an IOL and the corneal endothelium—whether in a transplanted or native cornea—poses a significant disadvantage, increasing the risk of endothelial cell loss and graft failure. By removing the anterior chamber IOL, we aimed to eliminate this source of mechanical stress and create better conditions for corneal graft survival.

Due to the absence of a capsular bag, we considered a scleral-fixated lens suspension using the flanging technique as the most elegant solution. In this case, we opted for the Yamane technique over Canabrava's double-flanged approach. This choice was guided by specific intraoperative conditions that favored the simplicity and efficiency of Yamane's method.

Firstly, the Yamane technique requires the creation of only two scleral tunnels compared to the four necessitated by Canabrava's approach. Reducing the number of tunnels minimizes surgical trauma and decreases the likelihood of intraoperative complications, particularly in the context of hypotensive conditions caused by the open-sky configuration. In these challenging circumstances, a less invasive technique was preferable for better control of intraocular pressure and tissue handling.

Secondly, the Yamane technique offers a shorter surgical time, further contributing to reduced surgical risk. In open-sky surgery, minimizing the duration of anterior chamber exposure is critical to lowering the risk of endophthalmitis and other postoperative infections. This consideration was especially relevant for this case, where prolonged open-sky exposure could have exacerbated patient outcomes.

Overall, our decision was tailored to achieve an optimal balance between surgical complexity, intraoperative safety, and postoperative prognosis.

To further contextualize the advantages of the Saloon Door Technique, we provide a comparative summary of key parameters versus alternative approaches (Table 1).

**Table 1:** Comparative overview of surgical approaches for IOL implantation with Artificial Iris (AI) management.

Parameter	AI-explantation + new AI with combined IOL implantation (Yamane technique)	AI-explantation + new AI-with combined IOL implantation (Canabrava technique)	Saloon Door Technique (Presented)
**AI Preservation**	Not preserved	Not preserved	Preserved
**Number of Scleral Passes**	2	4	2
**Surgical Time**	Significantly longer (++)	Significantly longer (+++)	Shorter (+)
**Invasiveness**	Higher (+++) AI explantation + AI + IOL implantation + 2 scleral tunnels	Higher (++++) AI explantation + AI + IOL implantation + 4 scleral tunnels	Lower (++) no AI removal – only IOL implantation + 2 scleral tunnels
**Risk of AI Dislocation/Tilt**	additional risk (+++) 2-point fixation may result in less precise positioning	additional risk (++) 4-point fixation may allow more precise positioning	Low (+)Pre-existing AI left intact, but potential risk of AI shift during saloon door maneuver
**Cosmetic Preservation**	Not applicable	Not applicable	Preserved
**Technical Difficulty**	High (+++)	Very High (++++)	Moderate (++) relative to alternatives

As outlined in Table 1, approaches involving explantation of the AI, such as AI removal followed by Yamane or Canabrava techniques, typically require more surgical steps, greater manipulation, and longer operative times. In contrast, the Saloon Door Technique preserves the existing AI, reduces trauma, and simplifies intraoperative management while maintaining stability and cosmetic outcome.

The most delicate part of the surgery was certainly the preparation of the scleral tunnel for subsequent externalization of the haptics. Due to the open sky situation and the fact that the eye had already been vitrectomized, the globe was virtually toneless and this made preparation considerably more difficult. The resistance of the sclera should not be underestimated here and the risks of iatrogenic trauma are naturally increased as a result. By filling the eye with BSS and simultaneously holding the bulb with forceps, the maneuver was successful. Additionally, the use of the forceps-needle proved advantageous in these challenging conditions, as it allowed direct grasping and externalization of the haptics without the need to feed them into a narrow needle lumen. This not only reduced the risk of losing or distorting the haptics but also minimized unnecessary manipulation of delicate ocular structures. Using disposable instruments, which maintain sharpness and precision, further reduced the risk of iatrogenic trauma associated with scleral tunnel preparation.

The incision of the AI with scissors was well managed, and we would recommend not cutting through the implant completely, but instead performing two controlled radial incisions. This technique preserves sufficient structural integrity to allow the AI to function as a flexible “saloon door,” enabling safe IOL passage without destabilizing the prosthesis. Although a foldable 3-piece IOL was used, implantation behind an intact AI was not feasible for several reasons. Firstly, the AI had been previously sutured into place, and manipulation through the small fixed pupil (approximately 3.35 mm) would have posed a significant risk of suture breakage or displacement of the iris. Secondly, once the first haptic is externalized during the Yamane technique, folding the IOL or inserting it through the non-incised pupil becomes technically impossible. The radial AI incisions enabled the IOL to be maneuvered safely into position and temporarily supported on the AI during externalization, reducing the risk of dislocation into the vitreous cavity — particularly important in an open-sky, vitrectomized eye. In addition, the radial orientation of the incisions helps distribute mechanical stress more evenly across the implant during IOL manipulation, minimizing the risk of tearing or material deformation. The cuts were limited in length and depth to preserve circumferential integrity and prevent structural compromise of the AI. Throughout follow-up, no signs of AI instability, decentration, or deformation were observed, supporting the safety of this approach.

While the Saloon Door Technique preserves the AI and avoids explantation, it should be noted that the radial incision represents an off-label use of the CE-marked implant. Although no postoperative instability or cosmetic issues were observed in this case, the long-term structural behavior of an incised AI is not yet fully known. Patients should be informed preoperatively that this technique deviates from the manufacturer's intended use and carries a theoretical risk of implant fatigue or deformation over time. Further studies would be needed to fully assess the long-term safety of this approach.

The patient had presented to our hospital after complicated cataract surgery and subsequent multiple corneal operations for the first time. Therefore, we do not have reports about pre-complicated cataract surgery and a CDVA before cataract surgery that could give us an indication of the severity of the amblyopia. Despite a relatively modest CDVA of +0.80 logMAR, the patient was subjectively satisfied, which is consistent with expectations given the presumed pre-existing amblyopia. It should be noted that the corneal suture removal was still pending at this time – an increase in visual acuity could still be expected after suture removal.

This case also underscores the psychological benefit, as the patient described the repeated ocular surgeries—particularly the recurrent corneal procedures—as a significant burden. The patient now feels greatly relieved, knowing that no further surgeries on the operated eye are anticipated in the near future.

## 6 Conclusion

In conclusion, the Saloon Door Technique for managing the pre-existing AI during scleral fixation presents a novel and effective solution to complex surgical scenarios. By making controlled incisions in the AI rather than removing it completely, this method facilitates the careful maneuvering of the IOL through the incised AI, while preserving its integrity and enabling safe flanging of the IOL haptics. This approach minimizes potential trauma to the surrounding structures, allowing for more precise and stable fixation of the IOL. Combined with the Yamane technique, this method not only enhances intraoperative control but also reduces surgical complexity. The Saloon Door Technique provides a promising and efficient option for managing cases with a pre-existing AI, offering a valuable addition to the armamentarium of modern anterior segment surgery.

**Value statement/highlights**

What was known:

• IOL exchange in cases with a pre-existing artificial iris (AI) typically requires removal or extensive manipulation of the iris
• Scleral fixation techniques, such as the Yamane technique, are effective for IOL implantation but can present challenges when dealing with AI devices

What this paper adds:

• This new technique allows IOL exchange behind an AI without its removal.
• By making controlled incisions in the AI, the IOL can be carefully maneuvered through the AI, preserving the integrity of the AI while enabling successful flanging of the IOL haptics

## CRediT authorship contribution statement

**Kilian Roth:** Writing – original draft, Resources, Investigation. **Konstantin Seiller-Tarbuk:** Visualization. **Michael Amon:** Writing – review & editing, Supervision.

## Patient consent

This report does not contain any personal information that could lead to the identification of the patient.

## Authorship

All authors attest that they meet the current ICMJE criteria for Authorship.

## Declaration of generative AI and AI-assisted technologies in the writing process

During the preparation of this work the authors used *ChatGPT by OpenAI* in order to improve language and readability. After using this tool/service, the authors reviewed and edited the content as needed and takes full responsibility for the content of the publication.

## Funding

No funding or grant support

## Declaration of competing interest

The authors declare that they have no known competing financial interests or personal relationships that could have appeared to influence the work reported in this paper.

## References

[1] Yamane S., Sato S., Maruyama-Inoue M., Kadonosono K. (2017). Flanged intrascleral intraocular lens fixation with double-needle technique. Ophthalmology.

[2] Canabrava S., Canêdo A.C., Lima D., Ribeiro G. (2019). Four-flanged intrascleral intraocular lens fixation technique: no flaps, no knots, no glue. Cornea.

[3] Muth D.R., Priglinger S.G., Shajari M., Kreutzer T.C., Mayer W.J. (2022). Novel surgical technique of sutureless artificial iris and intraocular lens scleral fixation using Yamane technique. Am J Ophthalmol Case Rep.

[4] Roth K., Amon M. (2025). Combined artificial iris and intraocular lens double-flanged scleral fixation in a patient with posttraumatic aniridia and aphakia. J Cataract Refract Surg Online Case Rep.

[5] Amon M., Bernhart C., Geitzenauer W., Kahraman G. (2021). The forceps-needle: combining needle and grasping functions in a single instrument. J Cataract Refract Surg.

