# Supplementary material for: Saloon Door Technique – “open sky” IOL exchange utilising flanged haptic fixation behind a pre-existing Artificial Iris
Source: Am J Ophthalmol Case Rep. 2025 Sep 10;40:102431. doi: 10.1016/j.ajoc.2025.102431 (PMC12466138; doi:10.1016/j.ajoc.2025.102431)
Supplement: Multimedia component 1 [file mmc1.pdf]

Supplementary Tab. 1: Postoperative Medication Regimen

Time Period	Dexamethasone 1mg/ml (preservative-free eye drops)	Prednisolone Pivalate 0.5mg/g (eye ointment)	Ofloxacin 3mg/ml (preservative-free eye drops)	Lubricants
Day 0–7	Every 2 hrs	Once nightly	4× daily	As needed
Day 8–30	4× daily	Once nightly	—	As needed
Month 2–9	Once daily	—	—	As needed
